# Thermal effect on the compression coefficient of heavy oil reservoir rocks

**DOI:** 10.1098/rsos.180534

**Published:** 2018-07-18

**Authors:** Zhang Chao, Yan Chuanliang, Liu Yuwen, Li Yang, Cheng Yuanfang, Tian Ji

**Affiliations:** 1 School of Petroleum Engineering, China University of Petroleum (East China), Qingdao 266580, People's Republic of China; 2 State Key Laboratory of Offshore Oil Exploitation, CNOOC Research Institute, Beijing 100028, People's Republic of China

**Keywords:** temperature, compression coefficient, porosity, heavy oil reservoir

## Abstract

The ever-decreasing oil resources receive more and more attention for the exploration and development of heavy oil reservoirs. Owing to the high viscosity and poor fluidity of heavy oil, it is necessary to use the method of injecting high-temperature fluid in the development process. But, this will cause a significant increase in the temperature in oil reservoir, and thus the compression coefficient of reservoir rock has a greater impact. The compression coefficient of heavy oil reservoirs at different temperatures was tested. The results show that the compression coefficient of rock is closely related to the nature of rock itself and its stress and temperature environment: the compression coefficient increases with the increase in rock porosity; the compression coefficient decreases with the increase in the effective confining pressure and increases with the increase in temperature. When the temperature is low, the increase in the compression coefficient is larger. As the temperature increases, the increase in the compression coefficient tends to decrease gradually. Because the temperature of the reservoir is higher than that of the ground, the influence of the temperature on the reservoir compression coefficient should be taken into account when carrying out the production forecast.

## 1. Introduction

With the increasing demands for energy resources and the declining of oil resources, more and more attention is paid to the exploration and development of heavy oil reservoirs. Owing to the high density, viscosity and poor mobility of heavy oils, it is difficult to find recovery rate with conventional development methods [1,2]. Thermal recovery methods need to be used in the production to improve the recovery efficiency of heavy oil reservoirs. At present, steam stimulation is an effective thermal recovery method for heavy oils [3]. In steam stimulation, the oil field is produced by depleted development after the end of shut in well. The value of reservoir compression coefficient is significant for the improvement of heavy oil recovery. Compression coefficient is an important parameter which reflects that the elastic energy can be provided by reservoir rocks, and high rock compression coefficient indicates sufficient elastic driving energy of oil reservoirs with which oil reservoirs can be easily exploited.

At present, empirical formula methods and experimental measurement methods are generally used to determine rock compression coefficient. The former mainly includes Hall's plot method and Newman's empirical formula method. Hall's plot method put forth the incorrect logical relationship that rock compression coefficient decreases with the increase of porosity and ignores the different compressibilities of different lithologies [4,5]. Newman's method considers only the relationship between rock compression coefficient and single factors, so the operating errors are large [6,7]. However, the latter obtains rock compression coefficient mainly by measuring the fluid volume flowing out due to the changes in pore volume under certain confining and pore pressure [8]. Based on the experiment, Rhett & Teufel [9] analysed the effect of reservoir stress path on compression coefficient of sandstones. Jalalh [10] analysed the influence of lithology and rock porosity on the compression coefficient of rocks by experiment. Based on the regression analysis on the test results of multiple groups of rock samples, Wang *et al.* [11] obtained the change laws of rock compression coefficient based on the compression processes and concepts of rock compression coefficient. Liu *et al.* [12] analysed the change laws of rock compression coefficient based on the experiment and studied the factors influencing the changes of rock compression coefficient. In addition, by using the experimental method, Ayoub & Bijan [13] researched the relationship between stress changes and rock compression coefficient.

However, the current research on the compression coefficient of rock is mainly carried out at room temperature, and the reservoir temperature is much higher than room temperature, especially for the thermal recovery reservoirs, the injection of high-temperature fluid will significantly increase the temperature of the reservoir. The temperature change can affect the mechanical characteristics of rock [14–18] such as Young's modulus and Poisson's ratio, but there is a lack of research on the effect of temperature variation on rock compression coefficient. A few studies have focused on the influence of high temperature on the compression coefficient of rock [19,20], and these studies have been conducted on compact rocks with low porosity. There is a lack of research on the compression coefficient of unconsolidated sandstones as heavy oil reservoirs. The cemented forms and microscopic structures of unconsolidated sandstones and compact rocks are very different. In order to study the thermal effect on compression coefficient of heavy oil reservoirs in the process of steam stimulation, the authors carried out experiments on compression coefficient in different temperatures.

## 2. Definition of rock compression coefficient

**Figure 1.**
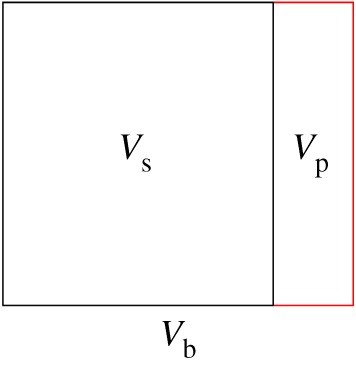
Volume relationship of rock.

The rocks have three kinds of volumes: the skeletal volume $V_{s}$, the pore volume $V_{p}$ and the apparent volume $V_{b}$ (figure 1), which meet the following relationship:

2.1 $$V_{b}=V_{p}+V_{s}.$$

**Figure 2.**
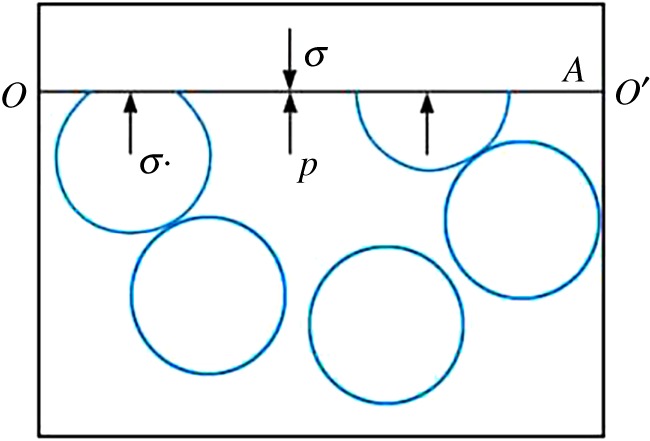
Stress relationship of rock.

The rock is subject to three stresses: skeletal stress $\sigma_{s}$, pore pressure *p* and external stress σ [21,22] (figure 2), and satisfies the following relationship:

2.2 $$\sigma =\sigma_{s}+\alpha P.$$

In the oil and gas production, due to the gradual extraction of fluids in pores, the pore pressure in reservoirs will decrease and the effective stresses will increase, which result in the gradual compression of reservoirs. As reservoirs are compressed, the reduction of pore volume squeezes the fluids in pores, thus increasing the production of oil and gas. Therefore, only the changes in pore volume with pore pressure are concerned in oil and gas engineering, so traditionally the pore volume compression coefficient of rocks is named as rock compression coefficient in oil and gas engineering. Compression coefficient of pore volume, that is, rock compression coefficient is defined as the change of unit pore volume when unit pore pressure is changed [11], and expressed as

2.3 $$C_{p}=\frac{\Delta V_{p}}{\Delta P}=-\frac{1}{V_{p}}\times \frac{\text{d}V_{p}}{\text{d}P},$$

where $C_{p}$ is the pore volume compression coefficient of rocks (simplified as rock compression coefficient below), (MPa^−1^). *P* and $V_{p}$ are the pore pressure (MPa) and the pore volume (cm^3^), respectively.

## 3. Test methods

**Figure 3.**
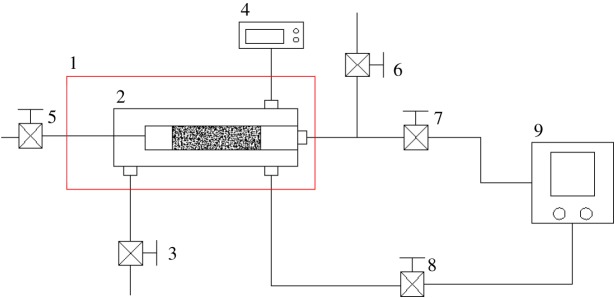
Flowchart of the experimental device for rock compression coefficient (1, heating device; 2, core holder; 3, vent valve for confining pressure; 4, temperature controller; 5, vent valve for pore pressure; 6, vacuum cut-off valve; 7, cut-off valve for pore pressure; 8, cut-off valve for confining pressure; 9, EDC servo control system).

In order to study the compression coefficient of reservoirs under high temperature and high pressure, an experimental device for measuring rock compression coefficient at high temperature and high pressure was established. This experimental device mainly included a high-temperature and high-pressure core holder, a confining pressure system, a pore pressure system, and temperature and pressure sensors. The flowchart of the device is shown in figure 3.

**Figure 4.**
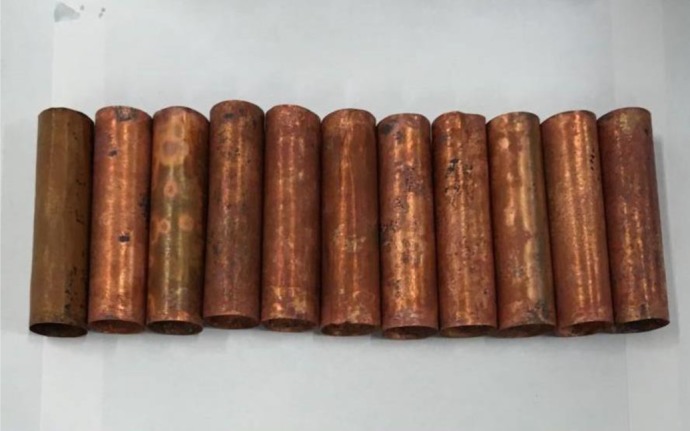
Copper casing.

The flange sealing structure was used in a core holder under high temperature and high pressure. The overall upstream and downstream plunger pistons were located in the chamber for confining pressure test, so that the end face of a rock was effectively contacted with the piston of the holder. Using the flexible graphite to seal the confining pressure in the rock samples and holder overcomes the shortage of traditional equipment that they cannot be applied for testing at high temperature when the rubber is used for sealing. Both ends of the core sample are connected to the indenters with a hole in the middle. The core and the indenter are wrapped in a copper casing and sealed so that all surfaces of the core are isolated from the confining pressure. Then the confining pressure can be effectively applied on the rock. The copper casing (figure 4) is made by turning a copper rod and then subjected to thermal treatment in the vacuum. The copper casing can work normally under the high pressure and high temperature because of a certain degree of toughness on the premise that it is thin and soft, which can prevent the copper casing from damage when the cores have distortion. The indenter has a hole in the middle that allows fluid to pass through, which is used to apply pore pressure to the core. The pore pressure and confining pressure were loaded by using the EDC servo control system, which can automatically control the pressure. When pore pressure or confining pressure changed, the servo control system was able to compensate pressure, so that pressure was maintained at a certain value and did not change with temperature or other conditions.

According to the oil and gas industry standard SY/T 5815-2008 (Test method of rock pore volume compressibility) [23] requirements of the People's Republic of China, the established compression coefficient measuring device is calibrated using water with a known compression coefficient, and the measurement error of the equipment does not exceed 5%.

**Figure 5.**
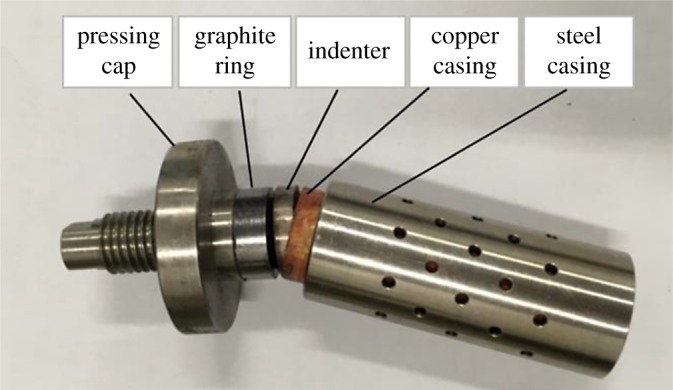
Schematic diagram of the sealing method of the core.

The sealing method of the core is shown in figure 5. In order to ensure the sealing of the ends of the copper casing, when the core is installed, the core, the indenter and the graphite ring are all put into the copper casing. The end surface of the indenter that contacts the graphite ring is a conical surface. And then the pressing cap is pressed downward on a mounting bracket, so that the graphite ring can closely fit with the indenter. At the same time, the graphite ring is compressed to expand laterally by the conical surface of the indenter. With the steel casing, the copper casing is pressed tightly to isolate the core from the confining pressure fluid.

This study measured the compression coefficient of rocks by measuring the changes of pore volume of rocks. In the measurement, the rock sample was placed in the envelope of the holder, and pore pressure and confining pressure were loaded in the sample and outside the envelope to stabilize the sample under confining pressure and pore pressure. After that, confining pressure was unchanged, while pore pressure would be decreased, so as to compress and decrease pore volume of the sample and force the fluids in pores to flow out. Through equation (2.3), the compression coefficient of rocks was calculated. When the test was finished, pore pressure reached the lowest and showed the maximum difference with confining pressure. Therefore, the difference between confining pressure and pore pressure at this moment was called the maximum effective confining pressure.

The pore pressure system was vacuumed for 4 h before the experiment. When the vacuum was applied, confining pressure was applied with 2 MPa. Confining pressure was used to tightly attach the copper casing to the core, to ensure that the deformation of the copper casing during the experiment is all due to the volume change of the core, and has no effect on the experimental results.

In order to prevent the temperature from rising too quickly in a short period of time, the liquid in the core holder rapidly expands, causing an overpressure hazard, the method of subsection heating is adopted. When the temperature reaches a certain value, heating is stopped, waiting for the pressure to stabilize before proceeding to the next step of heating. After the heating is completed, the pore pressure is reduced step by step after the pressure stabilized, and the pore volume under each effective confining pressure is measured.

## 4. Influences of porosity on the compression coefficient

By using the test system of the rock compression coefficient under high temperature and high pressure, the compression coefficient of man-made rock core of the heavy oil reservoir was determined. The sample in the test was obtained from a heavy oil reservoir with 1520–1560 m depth in Bohai Bay oil field, China.

**Figure 6.**
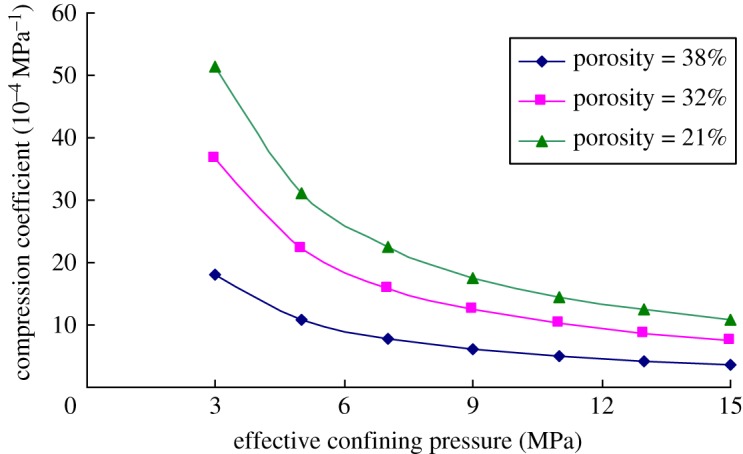
Variation of the compression coefficient of the rock cores showing different porosities with effective confining pressures.

Figure 6 shows the variation of the compression coefficient of the rock samples with the effective confining pressures at room temperature. It can be seen from the figure that the compression coefficient of rocks gradually decreased with the increase in effective confining pressure, and especially, the change trend was more obvious when the effective confining pressure was small. By taking the rock sample with the porosity of 21% as an example, when the effective confining pressure increased from 3 to 5 MPa, the compression coefficient of rocks reduced by 39.8%. While the pressure increased from 21 to 23 MPa, the compression coefficient of rocks decreased only by 8.6%. Such phenomenon is mainly because with the increase of effective confining pressure, the rock core was gradually compacted and the deformation resistance rose. Moreover, the pore space gradually reduced. When the pore space was compressed completely and disappeared, the compression coefficient became zero.

Furthermore, the compression coefficient increased with the increase in porosity of the sample. However, with the increasing of effective confining pressures, the difference in the compression coefficients with different porosities gradually decreased. This is mainly because deformation easily occurs to rock cores with a large porosity under the external loads due to the low cementation strength of the skeleton. As effective confining pressures increased, the rock core was gradually compacted and one with a large porosity was deformed more greatly. As a result, the difference between residual pore space of the rock core and that in rock cores with a small porosity gradually reduced, so that the difference in compression coefficients of the rock cores with different porosities gradually decreased with the increase in effective confining pressures.

## 5. Influences of temperature on the compression coefficient

**Figure 7.**
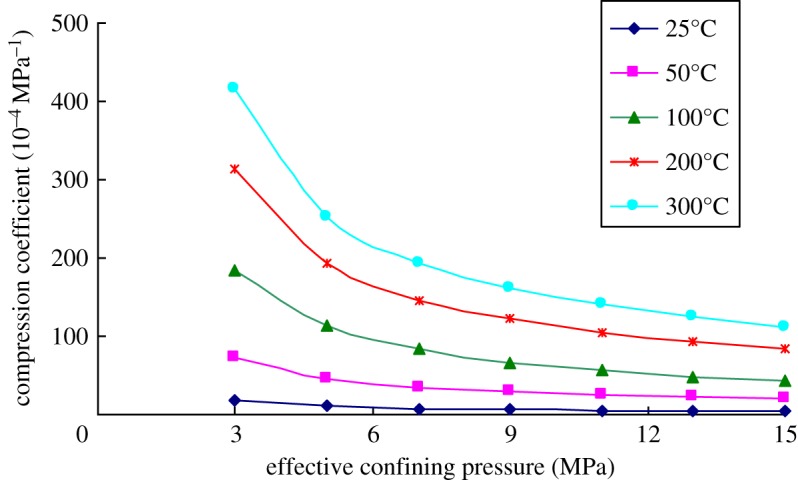
Variation of the compression coefficient with temperature (porosity = 21%).

**Figure 8.**
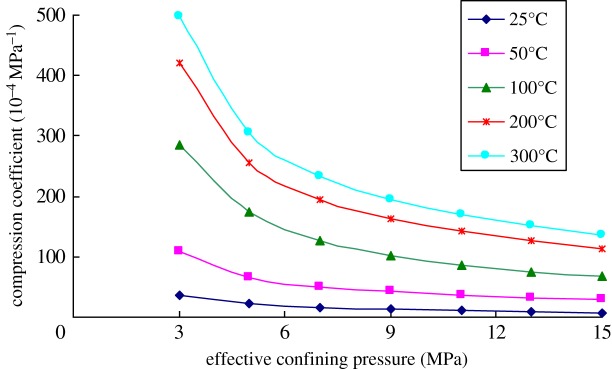
Variation of the compression coefficient with temperature (porosity = 32%).

**Figure 9.**
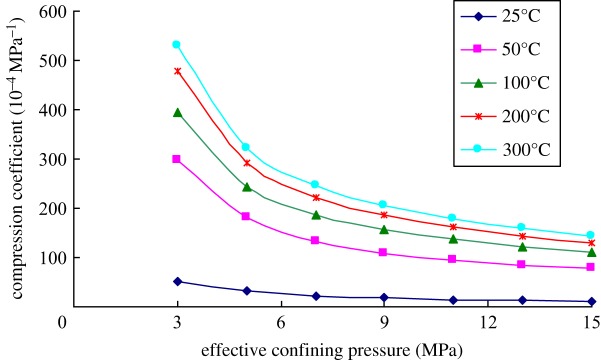
Variation of the compression coefficient with temperature (porosity = 38%).

Because heavy oil reservoirs need to be developed by using the thermal recovery, the compression coefficients of the rock cores collected from the reservoir were tested at different temperatures. Figures 7–9 show the variations of the compression coefficient of the rock samples with different porosities tested in different temperatures.

As shown in the experimental results, the compression coefficient of the rock core gradually increased with the increase in temperature, while reduced with the increase in effective confining pressures at any temperatures. Moreover, the difference in compression coefficients of the rock core at different temperatures gradually declined with the increase in effective confining pressures. This indicated that the influences of temperature on the compression coefficient gradually decreased with the increase in external loads.

**Figure 10.**
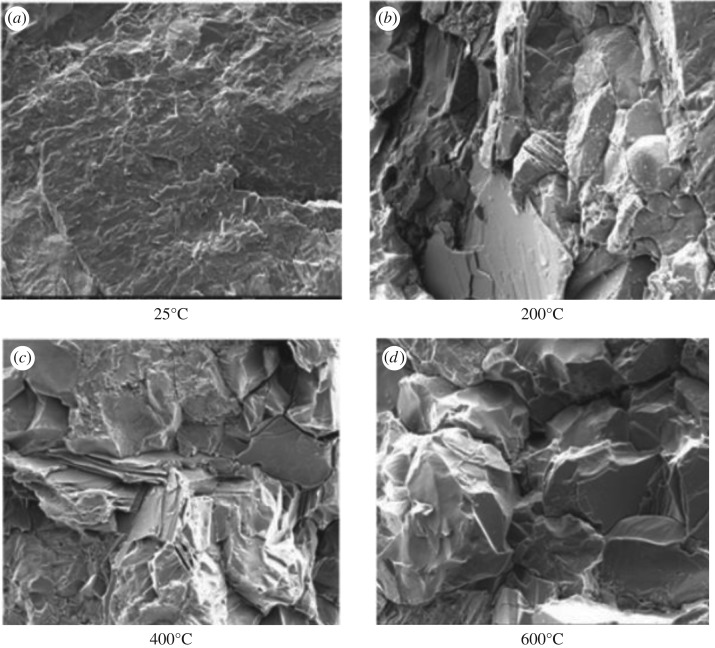
Morphology of sandstone at different temperatures [15]. (*a*) 25°C; (*b*) 200°C; (*c*) 400°C; (*d*) 600°C.

Rocks comprise different mineral particles which have distinct thermal expansion rates. Such difference in thermal expansion rate results in the expansion of internal cracks and induces the production and propagation of cracks at high temperature (figure 10) [15]. Furthermore, because of the increase in temperature, the stiffness of rock cement decreases and the slippage of particles increases [24–26]. Finally, the deformation resistance of rocks weakens and the compression coefficient rises.

**Figure 11.**
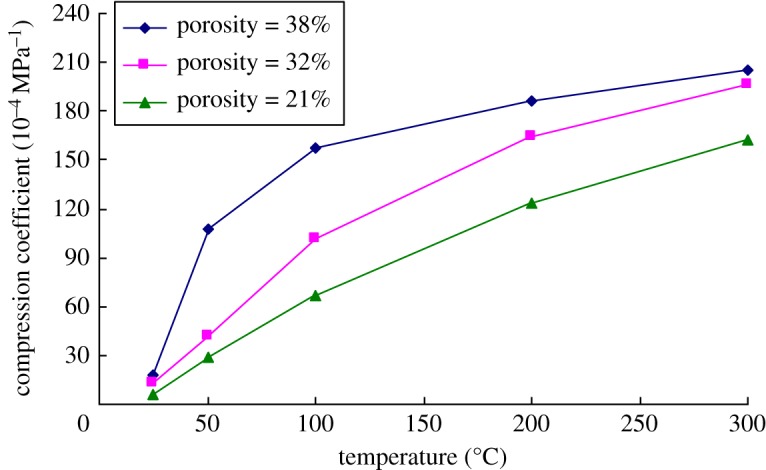
Impacts of temperature on the compression coefficient of rocks with different porosities (the effective confining pressure is 9 MPa).

Figure 11 is the variation of the compression coefficient of the rock cores with different porosities and temperature when the effective confining pressure is 9 MPa. It can be seen from the figure that there was difference in the increased amplitude of compression coefficient of rocks with temperature in different temperature ranges. When the temperature was low, the compression coefficient rose significantly. By gradually increasing the temperature, the increase in amplitude of compression coefficient gradually reduced. By using the rock sample with 38% porosity as an example, the compression coefficient of the rock sample increased by 140 × 10^−4^ MPa^−1^ when the temperature increased from 25 to 100°C. In comparison, as the temperature rose from 100 to 200°C, the compression coefficient of rocks increased only by 29 × 10^−4^ MPa^−1^. This indicated that the effects of temperature on compression coefficient of the heavy oil reservoir gradually reduced with the increase in the temperature.

At any temperature, the compression coefficient of the rock sample all gradually increased with the increasing porosity. As for the rock sample with a large porosity, the compression coefficient increased at an accelerating rate with the increase in the temperature at low temperature, while grew at a declining rate with further increase in the temperature. As to the rock core with a small porosity, the increase rate of the compression coefficient tended to be stable, although the increase rate was still faster at a low temperature, a small difference was found in various temperature ranges.

According to the relative increase amplitude, the temperature had greater effects on the compression coefficient of the rocks with a small porosity. When the temperature increased from 25°C to 300°C, the compression coefficient of the rock core with 21% porosity increased by 26 times, while that of the rock core with 38% porosity rose by 10.6 times. Therefore, for the heavy oil reservoir with a small porosity, the influences of temperature on the compression coefficient of the reservoir cannot be ignored.

When the thermal recovery was used in the heavy oil reservoir, the increase in temperature was beneficial for decreasing the viscosity of heavy oils and thereby reducing the flow resistance of heavy oils. Apart from this, raising the temperature also increases the compression coefficient of the reservoir, that is, the change rate of pore volume of rocks increases. And after the end of shut in well in steam stimulation, more crude oils are able to be driven into the well through the elastic energy of the reservoir, thus improving the productivity of oil wells. Therefore, while predicting the productivity of the thermal recovery for heavy oil reservoirs, the influences of temperature changes on the compression coefficient of reservoirs have to be fully considered.

## 6. Variation of reservoir compression coefficient around the well during steam stimulation

In order to analyse the variation of reservoir compression coefficient around the well during steam stimulation, the Abaqus finite-element package is selected for numerical simulation studies of the variation law of temperature field around the well in the process of steam stimulation. Abaqus is a general-purpose finite-element analysis code that can analyse fluid seepage and heat transfer problems in porous media and has been widely used for solving problems around a wellbore in recent years [27–33].

**Figure 12.**
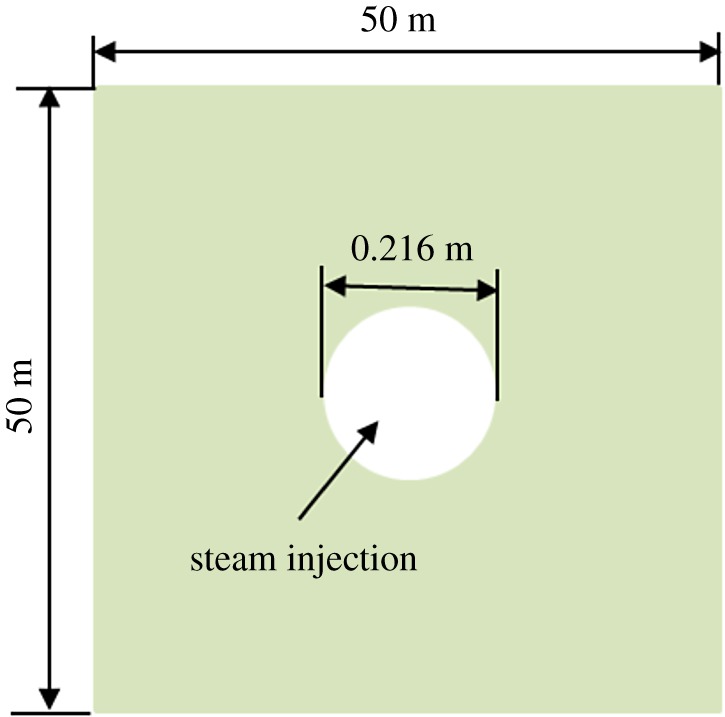
Schematic diagram of numerical calculation model.

This study mainly analyses the evolution law of reservoir compressibility at different distances from the wellbore during steam stimulation, and does not focus on the productivity of a reservoir. Therefore, a two-dimensional model (as shown in figure 12) is used to analyse the variation of the compression coefficient of the reservoir during the first cycle of steam stimulation. The model size is 50 × 50 m, and the borehole diameter is 0.216 m, which is located in the centre of the model. Around the model, fixed pore pressure and fixed temperature boundary conditions were added, and steam is injected into the wellbore. And the test results of rock compression coefficient with a porosity of 32% were used in the analysis. The material parameters are shown in table 1.

**Table 1.** Material parameters in the computational process.

parameter name	unit	value
rock density	kg m^−3^	2250
thermal conductivity of rock	J m^−1^ s^−1^ °C^−1^	2
thermal expansion coefficient of rock	°C^−1^	0.3 × 10^−6^
specific heat capacity of rock	J kg^−1^ °C^−1^	800
reservoir porosity		0.32
reservoir permeability	mD	1500
reservoir temperature	°C	50
steam temperature	°C	300
water density	kg m^−3^	1000
oil density	kg m^−3^	960
thermal conductivity of water	J m^−1^ s^−1^ °C^−1^	0.5
oil thermal conductivity	J m^−1^ s^−1^ °C^−1^	0.14
specific heat capacity of water	J kg^−1^ °C^−1^	4200
specific heat capacity of oil	J kg^−1^ °C^−1^	2000
thermal expansion coefficient of fluid	°C^−1^	0.3 × 10^−6^

### 6.1. Variation of compression coefficient in the stage of steam injection

**Figure 13.**
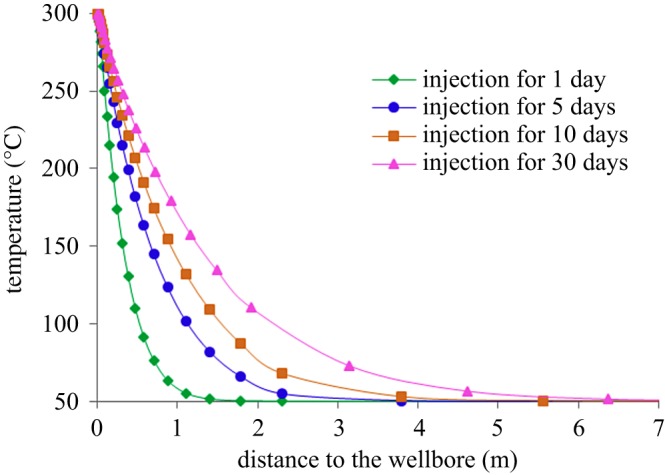
Variation of temperature field around the well in the stage of steam injection.

**Figure 14.**
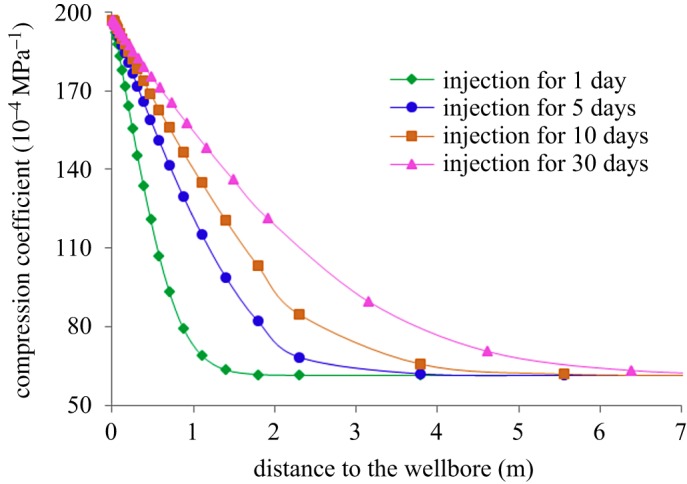
Variation of compression coefficient around the well in the stage of steam injection.

Figure 13 shows the variation of temperature field around the well in the stage of steam injection, and figure 14 shows the variation of reservoir compression coefficient around the well with injection time. During the process of steam injection, as the heat continues to spread from the well to the distance, the temperature around the well gradually increases, resulting in the increase in the compression coefficient of the reservoir. The compression coefficient is the largest at the wellbore and gradually decreases towards the reservoir. And as the injection time increases, the area with increasing compression coefficient also increases. Therefore, the injection of high-temperature fluid will greatly increase the elastic energy of reservoirs near the well, which is beneficial to enhance oil recovery during steam stimulation.

### 6.2. Variation of compression coefficient in the stage of production

**Figure 15.**
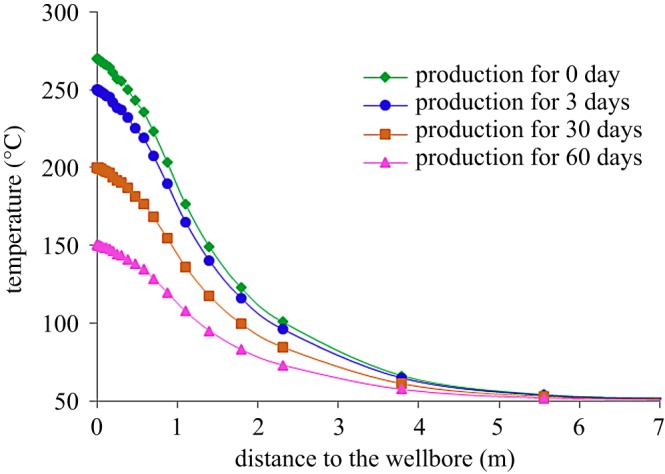
Variation of the temperature field around the well during production.

**Figure 16.**
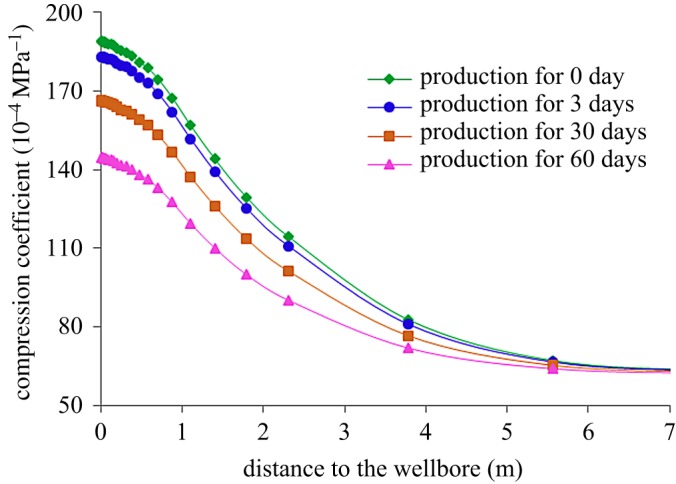
Variation of compression coefficient around the well during production.

After steam injection, with the increase in shut-in time, the heat in the heating zone will be transferred to the unheated zone. Therefore, the temperature in the heating zone will be reduced after shut-in well. Figure 15 shows the variation of temperature field around the well in the stage of production, and figure 16 shows the variation of reservoir compression coefficient around the well with production time. During the process of production, the temperature around the well gradually decreases due to the continuous recovery of high-temperature fluids, resulting in a decrease in the compression coefficient of the reservoir. At the same time, with the increase in production time, the temperature decrease range around the well gradually increases, and the elastic energy of reservoirs gradually decreases, results in the decrease in oil productivity.

## 7. Conclusion

At any temperature, the compression coefficient of the heavy oil reservoir gradually increased with the porosity of the reservoir. The compression coefficient of rocks gradually decreased with the increase in effective confining pressures, while the decrease rate reduced with the increase in effective confining pressures.

The compression coefficient of rocks in the heavy oil reservoir gradually rose with the temperature. When the temperature was low, the compression coefficients increased at a high rate. With the increase in the temperature, the increase rate of the compression coefficient gradually decreased. As for the rock with a large porosity, the increase rate of the compression coefficient with temperature was larger at a low temperature, while it rapidly decreased with the further increase in the temperature. For the rock core with a small porosity, the compression coefficient tended to increase at a stable rate and presented small difference in each temperature range.

The changes of the compression coefficient of reservoirs should be fully considered in the prediction of recovery effects of steam stimulation. Furthermore, because the temperature of oil and gas reservoirs is higher than that of ground, the influences of temperature on the compression coefficient should also be taken into consideration in the productivity prediction of other oil and gas reservoirs using depletion recovery method.

## Supplementary Material

Data for the experiment and calculation

## Data accessibility

The datasets supporting this article have been uploaded as part of the electronic supplementary material.
